# Integrated Proteomic Screening Reveals Heme Enzyme Depletion Induces Cyst-like Vacuole Formation in *Toxoplasma gondii*

**DOI:** 10.3390/ijms27188154

**Published:** 2026-09-13

**Authors:** Yafei Zhao, Yuanmeng Wang, Runyuan Yang, Aiyun Zhao, Zhenjie Zhang, Meng Qi, Hui Dong

**Affiliations:** 1 College of Animal Science and Technology, Tarim University, Alaer 843300, China; zyf101321@126.com (Y.Z.); wangyuanmeng@126.com (Y.W.); yry152133@163.com (R.Y.); zaydky@126.com (A.Z.); zhangzhenjie2019@126.com (Z.Z.); qimengdz@163.com (M.Q.); 2 Engineering Laboratory of Tarim Animal Diseases Diagnosis and Control, Xinjiang Production & Construction Corps, Alaer 843300, China

**Keywords:** *Toxoplasma gondii*, apicoplast, heme pathway, lytic cycle, large parasitophorous vacuole

## Abstract

The transport mechanisms for substrates and nutrients within the heme pathway of the *Toxoplasma gondii* apicoplast remain poorly understood. However, studies on heme metabolic enzymes have employed disparate genetic manipulation approaches, limiting direct phenotypic comparisons among different enzymes. This research involved screening potential apicoplast proteins in *Toxoplasma gondii* by cross-referencing and analyzing protein–protein interaction networks. Within the heme enzyme pathway of the apicoplast, eight enzymes were found to be predominantly conserved in the *Sarcocystide* family. Utilizing the CRISPR-Cas9 system alongside a U1 snRNP-mediated gene-silencing approach, we developed inducible knockdown strains—iKD-PBGD, iKD-UROS, and iKD-UROD—targeting three key metabolic enzymes crucial for the parasite lytic cycle, as demonstrated through replication experiments. To investigate the transport mechanisms for heme-related nutrients or substrates, we knocked down these three enzymes, using TgGRA12 as an initial marker. Continuous fluorescence signals highlighted the parasitophorous vacuole (PV) membrane surrounding tachyzoites during both early and late replication stages, particularly at 48 h post-rapamycin treatment, indicating a transformation of the cyst-like PV resembling that in *Toxoplasma gondii*. Phenotypically, knockdown of these heme enzymes led to the formation of slowly replicating, cyst-like parasitophorous vacuoles. However, this morphological change did not significantly affect the acute virulence of the parasites in vivo, as determined by mouse survival assays. This study explored the functional roles of the three intermediate metabolic enzymes, offering a novel viewpoint on the gradual demise of *Toxoplasma gondii* as a potential target for drug development.

## 1. Introduction

The phylum Apicomplexa is composed of Coccidia, including *Toxoplasma gondii*, and features the apicoplast organelle, a four-membrane compartment containing numerous metabolic pathways and essential biochemical reactions [1]. However, many unknown import and export transporters [2] sustained in parasite metabolic pathways that have been reported—such as TgFLP12 as a fatty acid transporter [3], TgAPC1 and TgAPC2 as pyruvate transporters [4], TgTPC as an apicoplast Ca^2+^ transporter, which can uptake and cause membrane contact site formation between the apicoplast and the ER (endoplasmic reticulum) [5], and TgAPT1 as a carbon skeleton transporter [6]—support these metabolism pathways. Numerous transporters remain to be characterized within the heme pathway of *Toxoplasma gondii* [2,7]. Putative heme-related transporters can be identified through the *T. gondii*^-Apico^ parasite database (ApiNeg-ARM/RH-MVA-ARM), which was established by genetic complementation with a four-gene mevalonate bypass cassette under supplemented conditions, including fatty acids, lysophosphatidic acid, 5-aminolevulinic acid, and mevalonolactone (MVL) [7].

In the context of the *T. gondii*^-Apico^ parasite model, proteomic studies [7], alongside the hyperLOPIT analysis of *Toxoplasma* proteomes [8] and APT1-TurboID proteomes [9], have identified numerous novel proteins within the apicoplast stroma (0 ≤ TMD < 6). Notably, the intermediate metabolic enzyme TgUROS was knocked out in the RH-MVA-HA-Apico strain, demonstrating that residual vesicles do not contribute to heme synthesis [7]. In the de novo heme biosynthesis pathway of *Toxoplasma gondii*, the knockout of TgALAS (first step, apicoplast), TgCPOX (sixth step, cytoplasm), and TgCPOX (seventh step, mitochondria) resulted in reductions in total heme levels by approximately 50%, 75%, and 15%, respectively. Additionally, the inducible knockdown of TgCPOX (eighth step, mitochondria) led to a nearly 15% decrease in total heme abundance [10]. Furthermore, the inducible knockdown of TgUROD (fifth step, apicoplast) resulted in an 80% reduction in heme levels, affecting the abundance of mitochondrial c-type cytochromes and cellular ATP levels [11]. The knockout of TgALAS, TgCPOX, and TgFECH impaired parasite survival, although this could be mitigated by the addition of 5-ALA. In contrast, the knockout of TgCPDH did not affect the virulence or quantity of cysts in the Me49 strain [12]. However, the impact of single inducible knockdowns of intermediate metabolic enzymes of the heme pathway, including TgPBGD (third step, apicoplast), TgUROS (fourth step, apicoplast), and TgUROD (fifth step, apicoplast), on parasite survival remains uncertain.

In the TCA cycle, acetyl-CoA serves as a substrate to produce succinyl-CoA, which aids in the synthesis of ALA from the mitochondria of *Toxoplasma gondii* to the apicoplast. Additionally, acetyl-CoA can generate the costly IPP from MVL when a four-gene mevalonate bypass cassette is genetically complemented [7]. The movement of endocytosed materials within *Toxoplasma gondii* has been documented through the prenylome proteome [13]. However, the roles of prenylated proteins in *Toxoplasma* related to heme nutrition and the transport of heme substrates via vesicle trafficking remain unclear. Notably, host cytosolic components, such as green fluorescent protein, can influence this trafficking through four specific proteins (TgRab1b, TgRab2c, TgRab5a, and TgRab7a) in *Toxoplasma gondii* tachyzoites [13]. The vesicles within the parasitophorous vacuole of *Toxoplasma gondii* are involved in either the translocation or uptake of materials across the interface [14,15] of the *Toxoplasma gondii* and host cell membranes.

Following the invasion of tachyzoites, *Toxoplasma gondii* GRA12 is secreted and forms a membranous tubulovesicular network that integrates into the parasitophorous vacuole at the anterior pole of the host cell [16]. TgGRA12 is present in both the soluble vacuolar fraction and the insoluble membranous fraction, functioning as both a soluble and transmembrane protein within the vacuolar environment [16]. Furthermore, TgGRA12 serves as a widespread virulence factor among various *Toxoplasma gondii* strains and different mouse subspecies. It plays a role in shielding the parasite from clearance mechanisms mediated by IFNγ. Recent studies have also identified potential host interactors, such as the murine ATPase plasma membrane Ca^2+^ transporting 1 and the mitochondrial protein Prohibitin 2, suggesting that TgGRA12 may facilitate interactions between the parasite and host cells [17].

The interaction between host cells and parasites occurs at membrane contact sites, which encompass various structures such as mitochondria-PV, endoplasmic reticulum-PV, plasma membrane-PV, Golgi-PV, cytosol-PV, and nucleus-PV [18]. These sites can be categorized into three types, with TgVIP1 at the *Toxoplasma gondii* Parasitophorous vacuole membrane (PVM) facilitating interactions between the endoplasmic reticulum and the PV through the host protein MOSD2, as well as VAPA and VAPB [19]. This process is associated with the canonical FFAT motif protein linked to TgROP1 [20]. Additionally, structures known as structures positive for outer mitochondrial membrane play a role in initiating TgMAF1 and the host protein TOM70, which is activated under conditions resembling chronic stress due to the involvement of proteins from both the inner mitochondrial membrane and outer mitochondrial membrane, including host proteins TOM20 and MFN2, located between the mitochondria-PV [21].

This research investigates the phenotypes of three heme-pathway intermediate enzymes in *Toxoplasma gondii* while also examining the role of dense granule proteins in the formation of parasitophorous vacuoles, with a focus on TgGRA12.

## 2. Results

### 2.1. Data Screening and Analysis of 23 Putative Toxoplasma gondii Apicoplast Stroma Proteome

**Figure 1:**
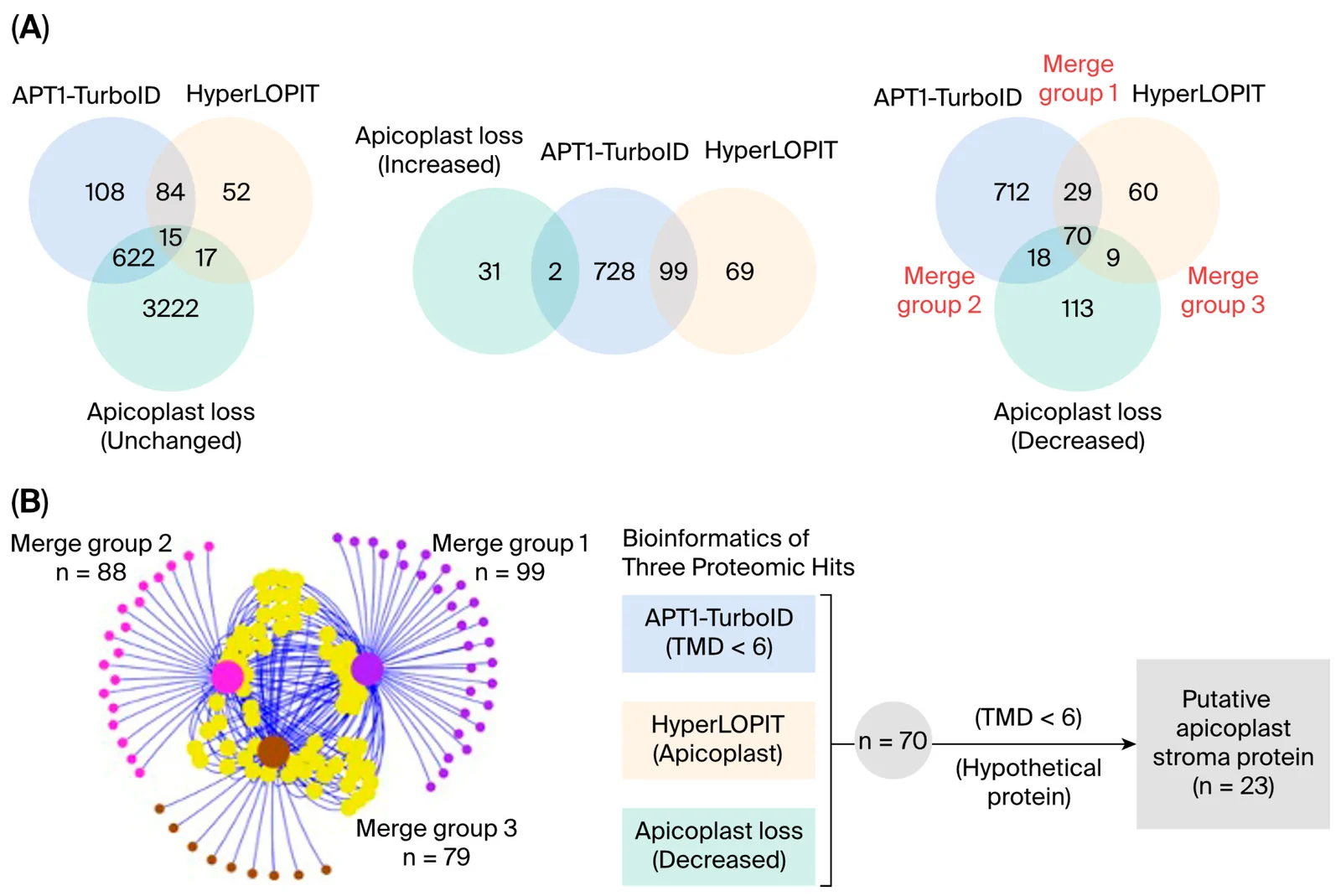
Analysis of the *Toxoplasma gondii* apicoplast proteome. (**A**) Venn diagram of APT1-TurboID [9] and HyperLOPIY-Apicoplast [8] cross with *T. gondii*^-Apico^ database (putative apicoplast proteins of unchanged, increased or decreased) [7], based on the web of Omicsmind (chttps://www.omicstudio.cn/home (accessed on 20 November 2025). (**B**) Diagram of 23 putative stroma apicoplast proteins screened by shared proteins among APT1-TurboID, HyperLOPIY-Apicoplast, and *T. gondii*^-Apico^ databases (putative apicoplast proteins of decreased) analyzed by Venn-based intersection analysis (https://www.bic.ac.cn/test/venn/#/ (accessed on 20 November 2025). Yellow circles indicate proteins shared among the intersected datasets, representing candidate apicoplast proteins predicted by set intersection rather than experimentally validated interactions.

A detailed subcellular atlas of the *Toxoplasma* proteome created using hyperLOPIT identifies nearly 128 proteins associated with the apicoplast membrane and apicoplast-soluble fractions. This includes their abundance distribution alongside endoplasmic reticulum proteins such as TgBip and TgHsp90 [8], as detailed in Table S1. Utilizing a biotin ligase tag, TurboID was endogenously fused with the membrane protein TgAPT1, revealing around 50 potential apicoplast or apicoplast-ER correlation proteins, constrained by a transmembrane domain (TMD ≥ 6). Meanwhile, the apicoplast stroma database is still under exploration for proteins with (0 ≤ TMD < 6) [9], as noted in Table S1. Additionally, the *T. gondii*^-Apico^ proteome database, referred to as ApiNeg-ARM/RH-MVA-ARM, has been presented, allowing for the identification of putative apicoplast proteins categorized as unchanged, increased, or decreased in the volcano plot data [7], also found in Table S1. This research aimed to leverage these three databases to chart the apicoplast stroma proteome of *Toxoplasma gondii*, illustrated in the Venn diagram in Figure 1A. The initial findings regarding apicoplast loss indicated that the *T. gondii*^-Apico^ database proteome remained unchanged, with 15 proteins highlighted in Figure 1A (Table S2). However, only two proteins overlapped with the *T. gondii*^-Apico^ database (notably increased) and the APT1-TurboID data (Figure 1A, Table S3). Importantly, a total of 70 proteins were identified across the three proteome groups (Figure 1A) (Table S4), forming a union of pairwise cross data that can be visualized in a Venn network (Evenn, https://doi.org/10.1002/imt2.184 (accessed on 20 November 2025)) [22] in Figure 1B. These findings can be further refined by screening for transmembrane domains and annotating hypothetical proteins (https://toxodb.org/toxo/ (accessed on 20 November 2025), ultimately narrowing down to approximately 23 putative apicoplast stroma proteins (Table S5).

### 2.2. Mapping Protein–Protein Interactions Among Screen Toxoplasma gondii Apicoplast Stroma Proteins

**Figure 2:**
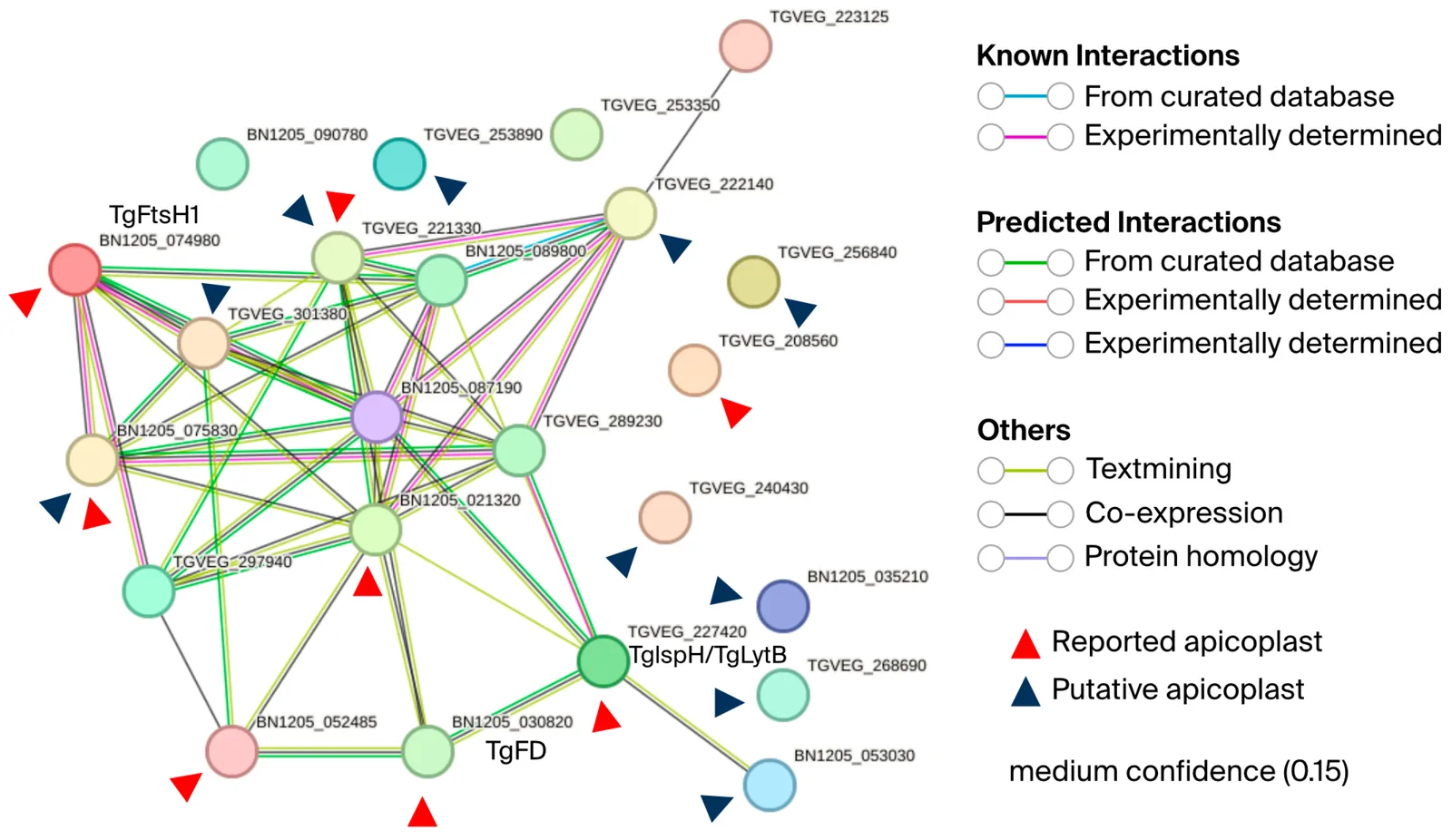
Protein–protein interaction network of putative apicoplast stroma protein (in *Toxoplasma gondii* or relevant organism). Network was constructed by STRING database (accessed on 23 December 2025) with a medium confidence score cutoff of 0.15. Putative apicoplast stroma proteins at central network have connection with each other like TgFD (BN1205_030820), TgIspH/TgLytB (TGVEG_227420), and TgFtsH1(BN1205_074980). Node colors represent distinct protein clusters or functional associations. Edge colors denote interaction evidence types: Known interactions include curated database entries (cyan) and experimentally validated interactions (magenta); predicted interactions include database predictions (green), experimental predictions (red), and other experimental evidence (blue); other associations include text mining (yellow-green), co-expression patterns (black), and protein homology (light purple). Triangular markers indicate subcellular localization: red triangles mark reported apicoplast-localized proteins, dark blue triangles indicate putative apicoplast proteins analyzed in this study. Protein identifiers follow the TGVEG (ToxoDB) and BN1205 nomenclature systems.

Following the examination of the unbiased interaction network, three proteomes comprising 70 shared proteins illustrate the protein–protein interaction dynamics, as shown in Figure S1 (Table S6). The sequences were sourced from ToxoDB and analyzed using STRING (https://cn.string-db.org/), with gene numbers converted to the TGVEG type III parasite, revealing sequence identities ranging from 88.90% to 100% at a medium confidence level of 0.4. Notably, the heme biosynthesis pathways TgUROS [7] and TgPBGS [12] have been highlighted in Figure S1 (Table S6). Additionally, to identify 23 potential apicoplast stroma proteins, sequence identities were again found to be between 88.90% and 100% (Table S6) at a medium confidence threshold of 0.15, as per STRING analysis. Among these proteins, TGGT1_201270 [23], TGGT1_239680 [23], TGGT1_264200 [7,10], TGGT1_211440 [8], and TGGT1_310770 [10] have been documented in the *Toxoplasma gondii* apicoplast. Furthermore, there are still nine putative apicoplast proteins that interact with metabolic pathway proteins such as TgFD [24], TgIspH/TgLytB [7], and TgFtsH1 [25] (Figure 2). Moreover, this research identified two potential apicoplast stroma proteins (TGGT1_306260, TGGT1_289760) that were screened in the APT1-TurboID database based on hypothetical protein annotations and transmembrane domains (0 ≤ TMD < 6). These proteins were found to colocalize with anti-IMC and anti-ACP, corroborating the findings of the HyperLOPIT comprehensive subcellular atlas (Figure S2) [8].

### 2.3. Toxoplasma gondii Heme Pathway-Related Enzymes Mainly Conserved in Sarcocystide

**Figure 3:**
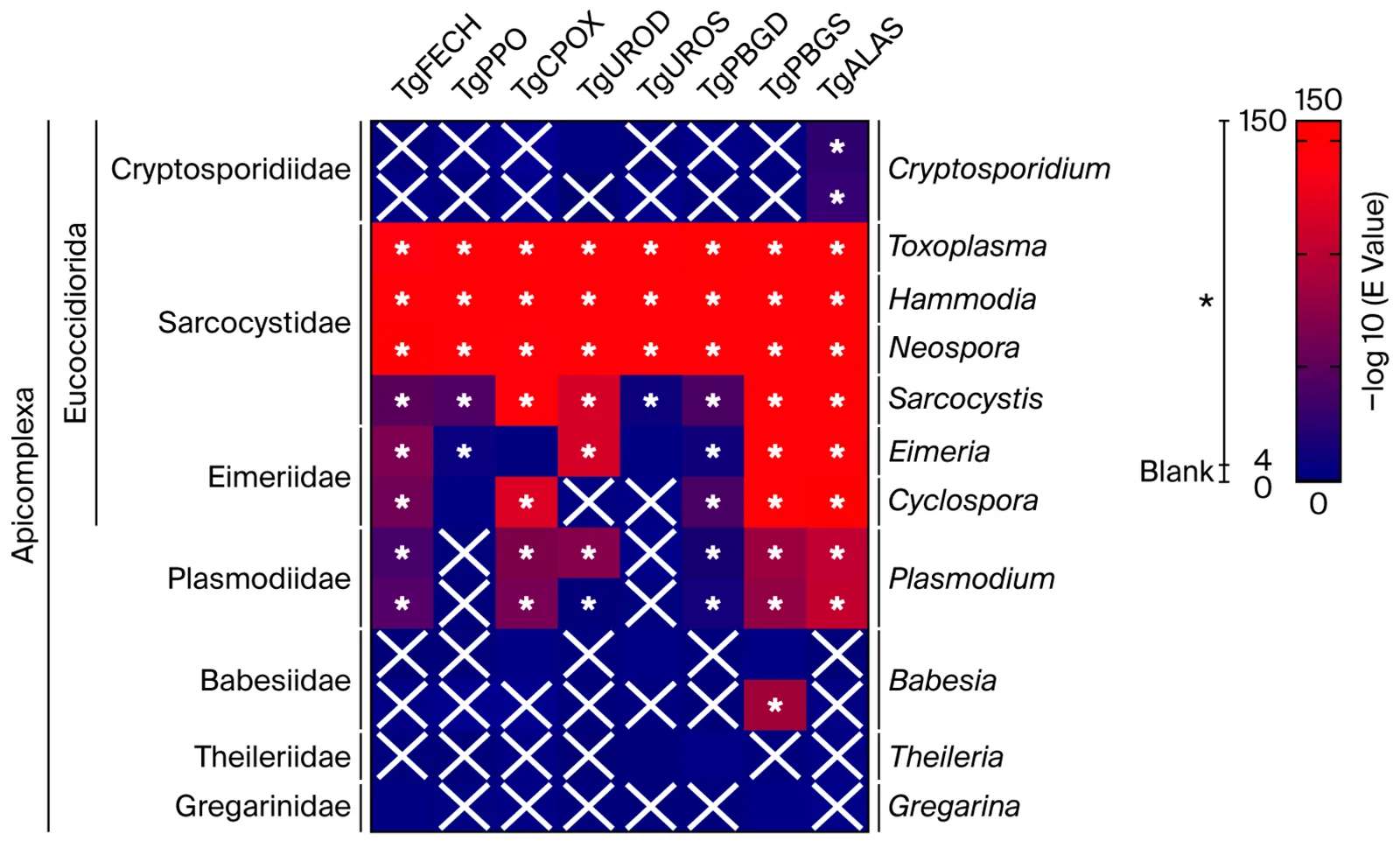
Homology analysis of heme biosynthesis pathway enzymes in apicomplexan. Heatmap showed homology significance of eight heme biosynthesis pathway enzymes (TgFECH, TgPPO, TgCPOX, TgUROD, TgUROS, TgPBGD, TgPBGS, TgALAS) in apicomplexan. The color gradient represents −log_10_(E-value), ranging from dark blue (0, no homology) to red (150, highly significant homology). White “×” indicates enzymes absent, while white “*” means homology [−log_10_(E-value): 4–150] [31,32]. Taxonomic groups are labeled on the left. The black “*” next to the color bar is a graphical marker used to link the color scale with the white “*” symbols in the heatmap, which represent detectable homology.

The analysis of four databases, including ToxoDB (https://toxodb.org/toxo/ (accessed on 19 November 2025)), PlasmaDB (https://plasmodb.org/plasmo/app/ (accessed on19 November 2025), PiroplasmaDB (https://piroplasmadb.org/piro/app/ (accessed on 19 November 2025), and CryptoDB (https://cryptodb.org/cryptodb/app (accessed on 19 November 2025), revealed a summary of the homology of heme pathway proteins (Table S7). Eight enzymes were found to be conserved within *Eucoccidiorida*, particularly TgALAs [12], which serve as the initial precursor in heme pathways. These pathways differ from those in primary heterotrophic eukaryotes and *Alphaproteobacteria*, which utilize the C4 pathway, while *Eubacteria*, *Archaea*, and phototrophic eukaryotes follow a series of reactions known as the C5 pathway [26,27]. Notably, *Chromera velia* exhibits differences in this context [26]. Although the oxygen-sensing protein TgCDPH is exclusively conserved in *Toxoplasma gondii* [12,28,29], there remain unidentified transporters [7] that facilitate substrate uptake [9] from host cells [27], indicating interactions between the apicoplast and mitochondria of *Toxoplasma gondii* [30], which differ in aerobic and anaerobic pathways [27]. This research indicates that the eight enzymes are most conserved among *Toxoplasma*, *Hammodia*, *Neospora*, and *Sarcocystis*, as illustrated in Figure 3. In *Eimeria tenella*, all but TgUROS and TgPPOX show high homology among the remaining six enzymes, while *Cyclospora cayetanensis* shares high homology with only five enzymes. Within *Eucoccidiorida*, TgALAS shows significant homology with *Cryptosporidium muris* and *Cryptosporidium andersoni*. Conversely, TgUROS and TgPPO lack homology with *Plasmodium falciparum* and *Plasmodium berghei ANKA*. Among *Babesiidae*, only TgPBGS exhibits homology with *Babesia bovis T2Bo*, excluding *Babesia ovata*, based on classification and analysis, contrasting with *Theileria* and *Gregarina*. These findings suggest that the enzymes involved in heme pathways are highly conserved throughout the evolution of these parasites.

### 2.4. Toxoplasma gondii Heme Pathway Where Three Intermediate Metabolic Enzymes Are Essential for Lytic Cycle

Conditional knockdown strains were developed using CRISPR-Cas9 to target three intermediate metabolic enzymes: TgPBGD, TgUROS, and TgUROD. An epitope tag (TY) was integrated at the endogenous locus, positioned after the stop codons of each enzyme, along with a U1 recognition site following the loxP sites. This was performed in a parasite strain that expresses dimerizable cre recombinase (DiCre strain) [30]. The primers for the iKD strains, detailed in Table S8, anneal within the gene loci and the hypoxanthine xanthine guanine phosphoribosyl transferase (HXGPRT) resistance cassette.

**Figure 4:**
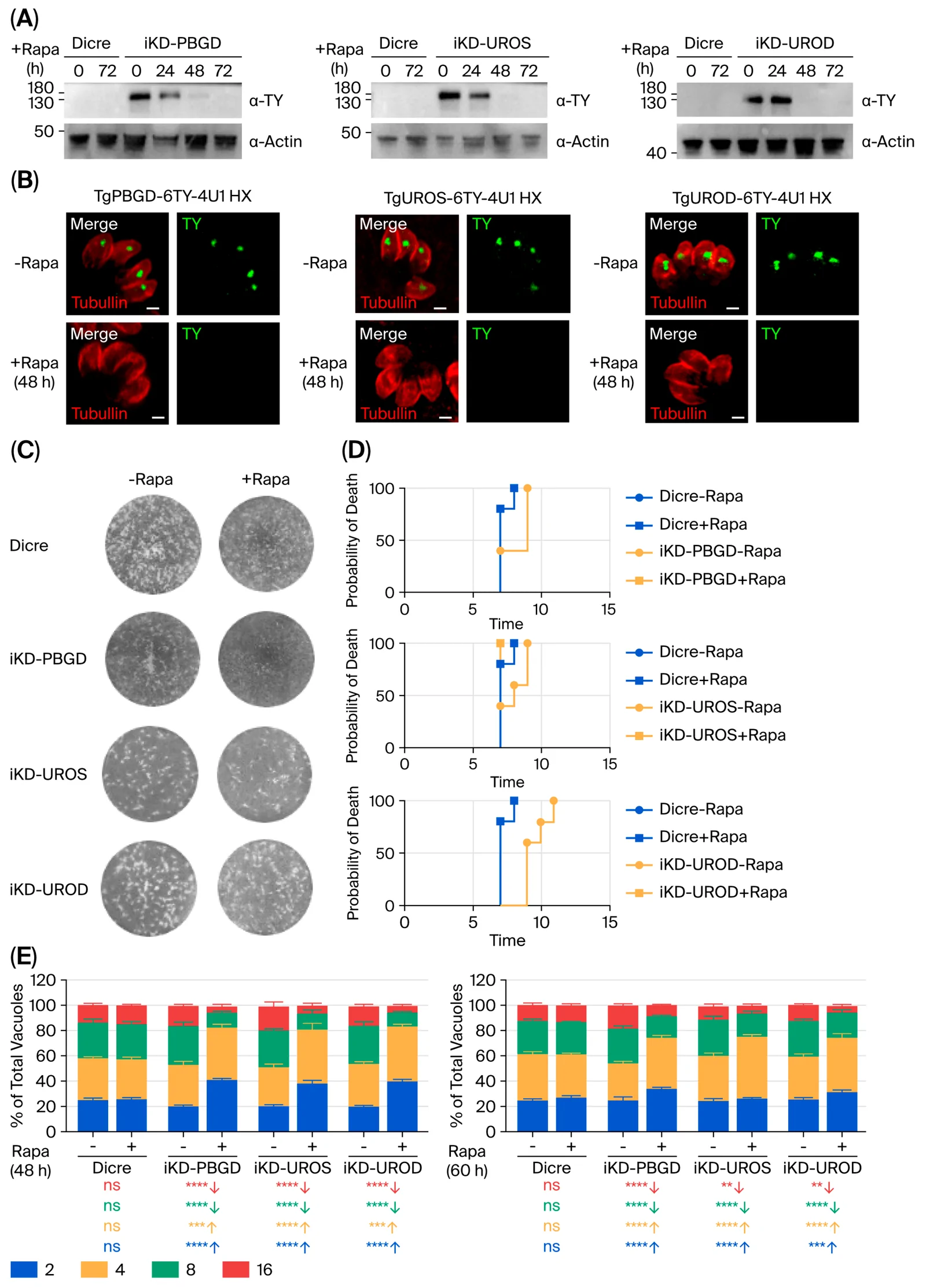
Inducible knockdown of three heme pathway intermediate metabolic enzymes results in plague, mice death, and large parasitophorous vacuolar formation. (**A**). Western blot of three heme pathway intermediate enzymes inducible knockdown (iKD) strains. DiCre strain with [33] targeted TgPBGD, TgUROS, and TgUROD with 6TY-4U1 [2] and strain treated with rapamycin (50 nM Rapa) for 0 h, 24 h, 48 h, and 72 h. Endogenous TY-tagged TgPBGD, TgUROS, and TgUROD expression was detected using anti-TY antibody, migrated about 84 KDa, 145KDa, 81 KDa, with anti-Actin as a loading control. The results show that protein levels of all three enzymes were significantly reduced within 24 to 48 h after rapamycin treatment. (**B**). Indirect immunofluorescence of three heme pathway intermediate metabolic enzymes inducible knockdown strains. iKD strains treated with rapamycin for 48 h, anti-Tubulin as parasite cytoskeleton marker, Scale bars = 10 µm. (**C**). Altered cellular morphology compared with plague experiment (Scale bars: 5 mm). DiCre, iKD-PBGD, iKD-UROS, and iKD-UROD strains were cultured in the absence (−Rapa) or presence (+Rapa) of Rapa. The single image shows approximately one well of a 6-well plate (well diameter ≈ 34.8 mm). (**D**). The probability of death compared to the viability of the parasite. Rapamycin-treated iKD-PBGD, iKD-UROS, and iKD-UROD strains showed no difference compared to control groups. The numbers of surviving mice (*n* = 5 per group) are indicated adjacent to the corresponding curves (e.g., 5/5, 4/5, 3/5, 2/5, 1/5, 0/5). (**E**) The replication of parasitophorous vacuoles at different stages. DiCre, iKD-PBGD, iKD-UROS, and iKD-UROD strains treated with rapamycin for 48 h or 60 h. Statistical significance was determined by two-way ANOVA: ns, not significant; ** *p* < 0.01, *** *p* < 0.001, **** *p* < 0.0001. Arrows indicate rapamycin-treated groups result in significantly increased or decreased numbers of parasitophorous vacuolar compared the rapamycin-untreated groups at each stage (tachyzoite numbers: 2, 4, 8, 16).

**Figure 5:**
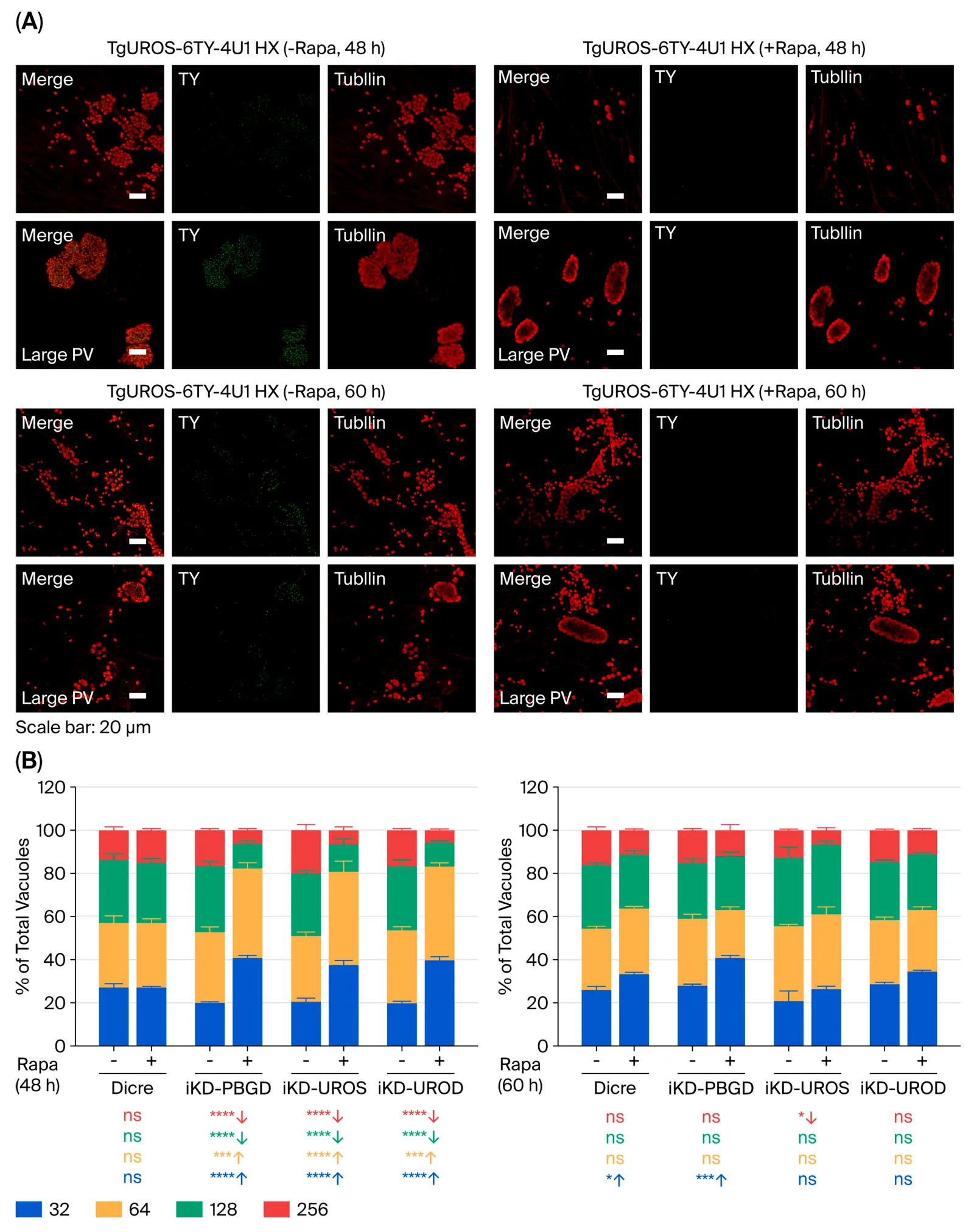
Indirect immunofluorescence and replication assay of large parasite vacuolar in three heme pathway intermediate metabolic enzymes inducible knockdown strains. (**A**). Indirect immunofluorescence of large parasite vacuolar in three heme pathway intermediate metabolic enzymes inducible knockdown strains. iKD strains treated with rapamycin for 48 h or 60 h, anti-Tubulin as parasite cytoskeleton marker. (**B**). The replication of intracellular vacuoles at different stages. DiCre, iKD-PBGD, iKD-UROS, and iKD-UROD strains treated with rapamycin for 48 h or 60 h. Statistical significance was determined by two-way ANOVA: ns, not significant; * *p* < 0.05, *** *p* < 0.001, **** *p* < 0.0001. Arrows indicate rapamycin-treated groups result in significantly increased or decreased numbers of parasite vacuolar compared the rapamycin-untreated groups at each stage (tachyzoite numbers: 32, 64, 128, 256).

Within the heme biosynthesis enzymes of *Toxoplasma gondii*, TgPBGD (TGGT1_271420, fitness score: −3.93), TgUROS (TGGT1_264200, fitness score: −4.88), and TgUROD (TGGT1_289940, fitness score: −4.6) have been identified and found in the apicoplast of *Toxoplasma gondii*, where they were endogenously tagged with three myc tags [10]. This research involved editing the loci of TgPBGD, TgUROS, and TgUROD using CRISPR/Cas9 in the DiCre strain, which expresses a dimerizable cre recombinase [33]. The knockdown of TgPBGD, TgUROS, and TgUROD was verified through Western blotting (WB) (Figure 4A) and indirect immunofluorescence assay (IFA) (Figure 4B). The U1 snRNP-mediated gene silencing system in DiCre-expressing parasites [34,35] resulted in varying effects on plague formation (Figure 4C) (Table S8), with the number and size of plagues indicating that TgPBGD, TgUROS, and TgUROD are crucial for the in vitro growth of tachyzoites. Notably, the knockdown of TgPBGD led to a more severe survival phenotype (Figure 4C) (Supplementary data—Original figures). In vivo knockdowns of TgPBGD, TgUROS, and TgUROD (Table S9), followed by intraperitoneal infection in mice, demonstrated that these genes did not significantly affect acute virulence of the parasite (Figure 4D), contrasting with the apicoplast membrane protein HAP9 (TGGT1_226790) [36]. Furthermore, replication studies indicated that the proliferation rates of TgPBGD, TgUROS, and TgUROD were altered when treated with rapamycin at 48 and 60 h (Figure 4E and Figure 5B).

### 2.5. Inhibition of Three Enzymes Involved in the Heme Pathway Led to a Notable Increase in Large PV Formation Primarily Observed at the 48 H Mark, While the TgGRA12 Fluorescence Signals Remained Consistently Unquantified

Under the IFA experiments of DiCre (Figure S3) and iKD-PBGD (Figure S4), iKD-UROS (Figure 5A), and iKD-UROD (Figure S5), tachyzoites increased (Normal PV, tachyzoites = 2 and 4; Large PV tachyzoites = 32 and 64) or decreased (Normal PV, tachyzoites = 8 and 16; Large PV tachyzoites = 128 and 256) with significant differences at the PV when rapamycin-treated under 48 h (Hours), PV-converted, or changed into cysts (Figure 4E and Figure 5B) [37]. At 60 h, tachyzoites only increased (Normal PV, tachyzoites = 2 and 4) or decreased (Normal PV, tachyzoites = 8 and 16) with significant differences at the PV when treated with rapamycin, with the DiCre strain as control (Figure 4E, Figure 5 and Figures S3–S5). Anti-GRA12, as a dense granule protein [37] associated with the intravacuolar membrane nanotubular network after the invasion, exists in both a soluble and a membrane-associated form [16].

**Figure 6:**
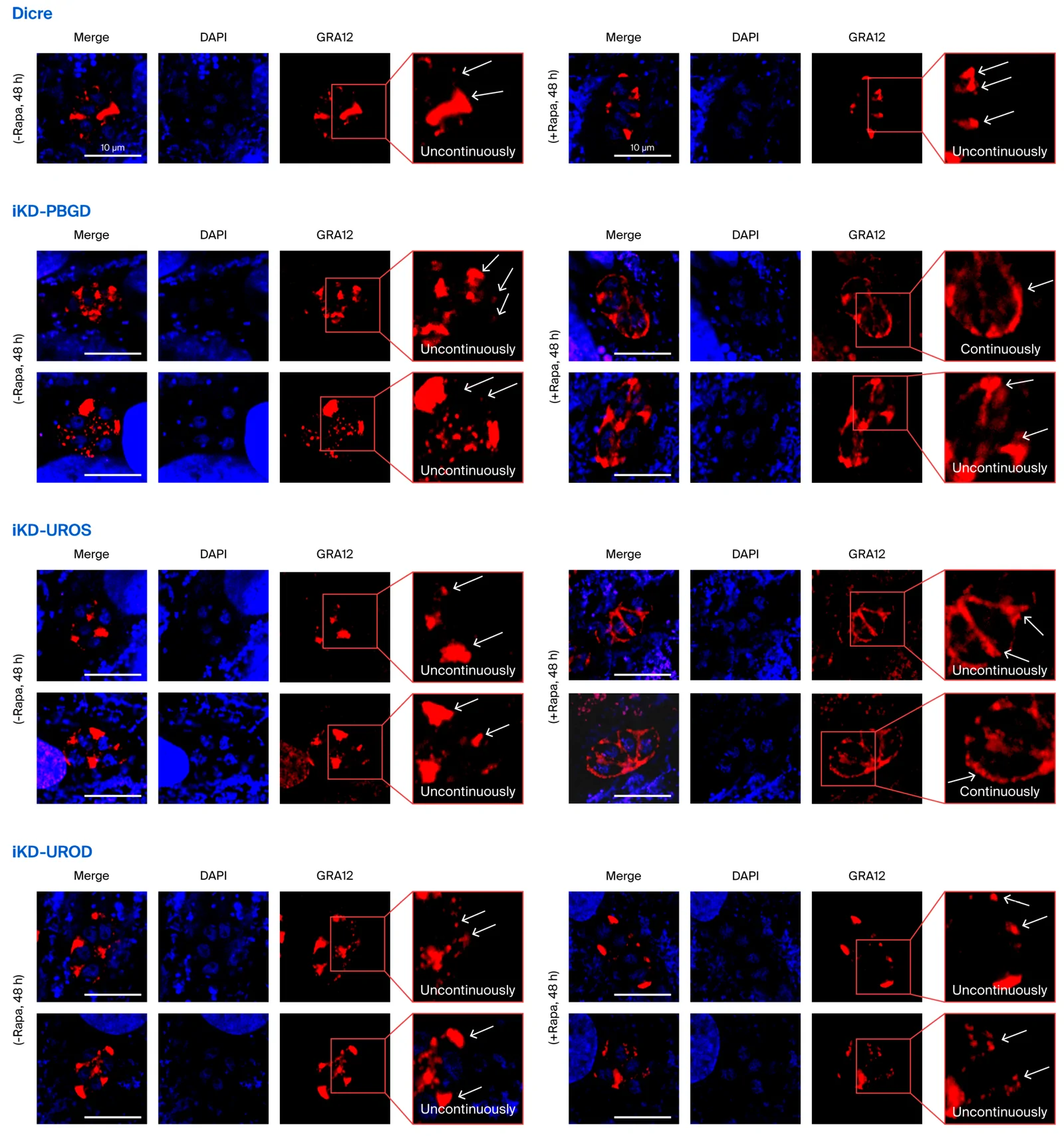
Indirect immunofluorescence assay of three heme pathway intermediate metabolic enzymes inducible knockdown strains. DiCre and iKD strains treated with rapamycin for 48 h, anti-GRA12 as parasite vacuolar marker. The arrow indicates the continuity of the fluorescence signal, Scale bar =10 µm.

**Figure 7:**
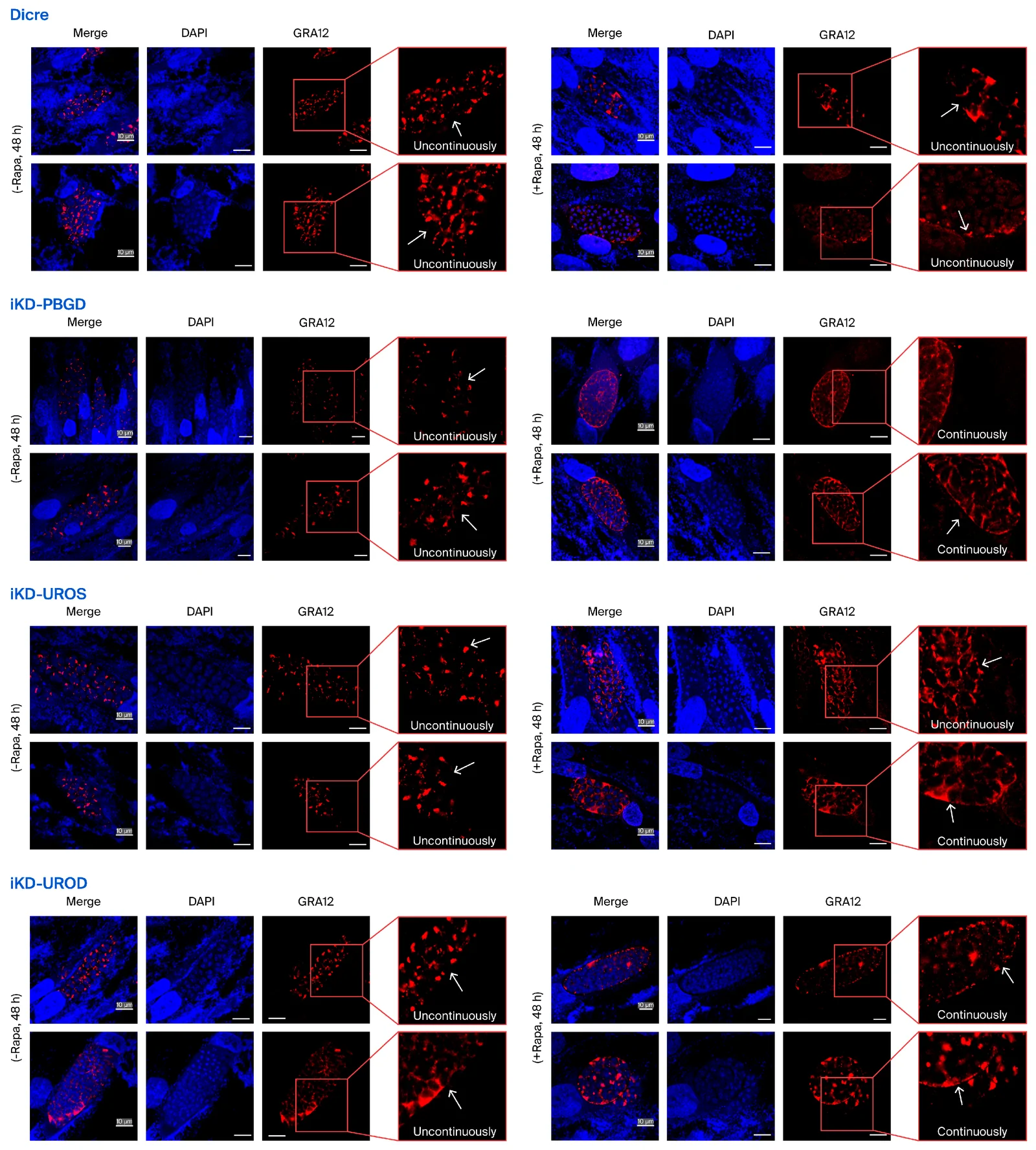
Indirect immunofluorescence of large parasite vacuolar in three heme pathway intermediate metabolic enzymes inducible knockdown (iKD) strains. DiCre and iKD strains treated with rapamycin for 48 h, anti-GRA12 as parasite vacuolar marker. The arrow indicates the continuity of the fluorescence signal, Scale bar =10 µm.

GRA12 localizes to the IVN [16] during acute infection, where it maintains functional integrity for nutrient uptake and material distribution. Genetic deletion of individual GRA12 family members [38] alters cyst burdens and reactivation efficiency, confirming their functional significance in cyst formation and persistence. Therefore, TgGRA12 was identified as a key marker, with persistent fluorescence signals observed [17], highlighting the parasitophorous vacuole membrane encasing tachyzoites during both the initial and later phases (Normal PV, tachyzoites = 2–16; Large PV, tachyzoites = 32–256) of replication, particularly at 48 h post-rapamycin treatment. This led to the transformation of the vacuole into a structure resembling *Toxoplasma gondii* cysts (Figure 6 and Figure 7) in the iKD-PBGD, iKD-UROS, and iKD-UROD strains, with the DiCre strain serving as a control. In contrast, the phenomenon of vacuole collapse was noted at successive time points following the knockout of TgGRA17, where small molecules facilitated the passage of dense granule proteins [39].

## 3. Discussion

*Toxoplasma gondii*, an apicomplexan parasite, is transmitted through food and can lead to widespread infections affecting various tissues, such as the brain and muscles. This organism has evolved from *Cyanobacteria* and transitioned into terrestrial animal hosts via primary and secondary endosymbiotic events. The apicoplast plays a crucial role in the metabolic processes of *Toxoplasma gondii*, a feature that is absent in *Cryptosporidium* [40].

Recently, the proteomes of *Toxoplasma gondii* (specifically the ME49 reference strain) and *Cryptosporidium* (*C. parvum* oocysts) were analyzed using hyperLOPIT, revealing insights into the localization of parasite cell fluorescence and the interactions of complex proteomes with host cells, as seen in *Toxoplasma gondii* [8]. Additionally, the study examined the orthologous proteins of *Cryptosporidium* in relation to other Apicomplexans, focusing on their cellular fluorescence localization and functions [41]. In *Toxoplasma gondii*, the protein TGME49_211440 (also known as TGGT1_211440, with a Fitness Score of −5.67) was identified in the RH strain and confirmed to be situated in the apicoplast using a streptavidin marker, contributing to a list of 168 apicoplast proteins [42]. However, other *Toxoplasma gondii* proteins with fluorescence localization predictions, such as those associated with the plasma membrane, endoplasmic reticulum, and mitochondrial membranes, have also been confirmed to reside in the apicoplast [9]. Among these, the uncharacterized TGME49_268690 is noted as a homolog of *Cryptosporidium parvum* cgd7_200, which is linked to small granules (a type of secretory organelle) and clusters of dense granules containing 23 proteins [41]. Furthermore, utilizing a homology gene number analysis method within Apicomplexans, this research directly transcribed gene names and conducted analyses to compile a selected proteome of apicoplast candidates [7,8,9] (Figure 1, Tables S1–S5). Nonetheless, three existing apicoplast databases were utilized, and information was filtered through cross-referencing methods to provide insights into proteome cross-linking, and further analyze a putative apicoplast protein network (Tables S2–S4).

In this study, two candidate proteins from the apicoplast stroma, TGGT1_306260 and TGGT1_289760, were identified through the APT1-TurboID database based on their descriptions and transmembrane domain characteristics (0 ≤ TMD < 6). Their colocalization with anti-ACP was confirmed by the HyperLOPIT comprehensive subcellular atlas (Supplement Figure S2) [8]. Among these, TGGT1_306260 is analyzed in InterPro and referred to as TgIspD or TgYgbP, which are cytidylyltransferases involved in the MEP pathway [42]. On the other hand, TGGT1_289760, also noted in InterPro, is identified as a biotin protein ligase that may play a role in the carbohydrate–pyruvate metabolism of the apicoplast alongside TgACC1, a known biotinylated protein [43]. Additionally, TgAT1 is implicated in carbohydrate metabolism [44].

Utilizing multi-proteome approaches or genome-wide screenings is a proficient way to identify target proteins. The engineered ascorbate peroxidase technique allows for the labeling of mitochondrial matrix proteomes within cells [45]. Notably, in *Toxoplasma gondii*, the GRA1 bait expressed as GRA1-BirA and GRA1-APEX has introduced a new method for protein discovery, leading to the identification of a novel dense granule protein [46]. Additionally, a homology protein from the inner mitochondrial membrane of mammalian cells, *Toxoplasma gondii* putative porphyrin transporter (Tgtmem14c), was discovered through a genome-wide screen conducted under 50 nM dihydroartemisinin. Similarly, the mitochondrial serine protease TgDegP2 was identified in a genome-wide screen under 0.5–10 μM dihydroartemisinin, suggesting that Tgtmem14c and TgDegP2 play roles in modulating artemisinin susceptibility in human foreskin fibroblasts when exposed to sublethal doses or lower concentrations of dihydroartemisinin [47].

Prior to this, the focus on metabolic pathways in the study of *Toxoplasma gondii* apicoplast has revealed the processes involved in the import and export of apicoplast proteins [2,48]. Investigations utilizing bioinformatics, such as mRNA abundance analysis [22] and biotinylation-based proteomics in malaria parasites [49], have contributed to these findings.

In examining the findings of this study, it is noted that the hypothetical apicoplast proteins (*n* = 23) exhibit a weaker association with TgUROS [7] compared to nine other putative apicoplast proteins, which are absent from the protein interaction network. These include proteins such as TGGT1_201270, TGGT1_239680, and TGGT1_310770 [23]. The research emphasizes the heme enzymes of *Toxoplasma gondii*, which share similarities with apicoplast membrane proteins: HAP18, HAP29, HAP36, and HAP46 [9]. The homology analysis reveals that eight heme enzymes of *Toxoplasma gondii* are conserved across coccidia, particularly in relation to *Hammodia* and *Neospora*, while showing lower similarity to *Gregarina*, *Theileria*, *Babesia*, *Plasmodium*, and *Cryptosporidium*.

The de novo heme synthesis pathway in *Toxoplasma gondii* comprises nine key enzymes, as noted by [12]. The initial reaction requires glycine and succinyl-CoA, facilitated by TgALA. It was observed that the Δalas::NLuc mutant, a *Toxoplasma gondii* strain expressing NanoLuc luciferase, exhibited a nearly 20% reduction in growth after 48 h of direct knockout, as indicated by NLuc intensity measurements in the mitochondria of *Toxoplasma gondii*. Additionally, the average size of the PV decreased by approximately 16% in the absence of ALA. In the sixth and seventh steps, the knockout of TgCPOX and TgPPO individually resulted in a 14% reduction in average PV size after 40 h. In vivo test demonstrated that mice survived up to the seventieth day. The eighth enzyme, TgFECH, when endogenously replaced and expressed using a hybrid promoter combining the Toxoplasma SAG4 promoter with an anhydrotetracycline (ATC)-responsive promoter, showed a knockdown effect at 48 and 144 h post-ATC treatment, leading to average PV reductions of nearly 10% and 32%, respectively [12]. Notably, *Toxoplasma gondii* undergoes endodyogeny, which encompasses the G1, S, G2/M/C, M, and C phases of the cell cycle [50], suggesting that the functions of heme-related enzymes vary across different phases of the cycle in *Toxoplasma gondii*.

This research highlights the critical role of three enzymes involved in the heme biosynthetic pathway during the lytic cycle of the parasite. Utilizing a U1 snRNP-mediated gene silencing approach via DiCre-expressed parasites, the expression levels of TgPBGD, TgUROS, and TgUROD were found to be diminished after 48 h. However, both in vivo and in vitro experiments indicated that this reduction did not significantly affect the parasite behavior. Notably, variations in PV counts were observed at 48 h and 60 h, with the potential for these vacuoles to develop into cyst-like structures containing up to 256 tachyzoites upon parasite release. This finding contrasts with previous studies on *Toxoplasma gondii* apicoplasts [8,11,51] and is also inconsistent with the roles of apicoplast membrane proteins TgAMT1 and TgAMT2 [9], as well as the proteins TgAPC1 and TgAPC2 [4].

To gain deeper insights into the formation of parasitophorous vacuoles, this research utilized *Toxoplasma gondii* TgGRA12 as a marker to observe changes in the development of the vacuole when treated with rapamycin. Continuous fluorescence signals from TgGRA12 were detected around the tachyzoites within the vacuole, particularly during the phase when four tachyzoites were present or in larger cyst-like vacuoles containing up to 128 tachyzoites (Figure 6 and Figure 7). The genes associated with TgGRA12 include TGME49_288650, TGME49_220890, TGME49_275860, TGME49_275850, and TGME49_308970, all of which possess a plectin domain akin to the hemidesmosomes found in various human cells [52]. The human plectin protein connects membrane proteins to keratin intermediate filaments, and this complex also includes a protein with an integrin domain, which can be explored using SWISS-MODEL (https://swissmodel.expasy.org/). This is similar to the analysis of the *Toxoplasma gondii* protein TGGT1_233220 (TgVIP1), which interacts with host proteins MOSD2, VAPA, and VAPB at the *Toxoplasma gondii* PVM [19]. Regarding heme transport, the phenotype of the fatty acid transporter TgFLP12 is influenced by varying culture conditions (1%, 10%, or 0% FBS), and the addition of 5-ALA or PPIX under 1% FBS does not alleviate the growth defect resulting from TgFLP12 depletion, suggesting that TgFLP12 is not involved in heme trafficking [3]. However, although the large PVs induced by heme enzyme depletion morphologically resemble tissue cysts, DBA staining did not confirm the presence of typical cyst wall components observed with confocal microscopy, suggesting that these structures represent slowly replicating, enlarged vacuoles rather than genuine bradyzoite cysts—a distinction that awaits future validation using bradyzoite-specific markers and type II strain backgrounds. This study addresses the biological rationale for selecting TgGRA12 as a marker and tracks the alterations in TgGRA12 fluorescence signals when the heme pathway enzymes of *Toxoplasma gondii* are silenced.

## 4. Materials and Methods

### 4.1. Parasite Strains and Cell Culture

The *Toxoplasma gondii* strains RHΔ*hxgprt*Δ*ku80* [9,53] and RHΔ*hxgprt*Δ*ku80*-T2A-DiCre (DiCre) [33,35,36] were obtained from Professor Shaojun Long at China Agricultural University. The human foreskin fibroblast cell line HFF-1 (ATCC SCRC-1041, approximately passage 19) [9] was also sourced from Professor Long, while another HFF-1 line (ATCC SCRC-1041, around passage 2) was purchased from Beijing Nang Cheng Co., Ltd. (BNCC) (Suzhou, Jiang Su, China). Plasmids and antibodies were requested from Professor Long at China Agricultural University [9,30,36], as listed in the Supplementary Material Table S10. The RHΔ*hxgprt*Δ*ku80* and DiCre strains were primarily cultured in HFF using Dulbecco Modified Eagle Medium supplemented with approximately 10% fetal bovine serum (TransGen, FS301-01 or FS301-02, Beijing, China), 2.38 g/L HEPES (Amresco, 7365-45-9, Solon, OH, USA), 3.7 g/L sodium bicarbonate (FUCHEN, 7365-45-9), 200 mM glutamine (Solarbio, G0200, Beijing, China), and an additional 100 mg/mL penicillin–streptomycin–gentamicin solution (Solarbio, P1410). The cell lines and strains were kept in a humidified incubator at around 37 °C with 5% CO_2_ to ensure optimal growth conditions.

### 4.2. Generation of Transgenic Strains

Endogenous tagged strains were created by integrating the epitope tag (6TY) into the native loci of the RHΔ*hxgprt*Δ*ku80* background strain using CRISPR-Cas9 techniques [54,55]. This integration occurred post-stop codons, along with the introduction of a homologous repair template for dihydrofolate reductase (DHFR) resistance within the native sequences. Inducible knockdown strains were developed by editing the DiCre strain [33], also positioned after stop codons. The templates were amplified using TransStart^®^ FastPfu Fly DNA Polymerase (TransGen, AP221-13). Transfected parasites underwent a screening process lasting over a week in a medium containing 25 µg/mL mycophenolic acid and 50 µg/mL xanthine, followed by clonal selection through serial dilution in 96-well plates, similar to the iKD strains. Electroporation was performed using SCIENT Z (SCIENTZ-2CS, SCIENTZ, Ningbo, China) and BIO-RAD (Gene Pulser Xcell, Bio-Rad Laboratories, Hercules, CA, USA) under two different conditions: one at 2500 V (Volt), 25 μF (Microfarad) capacitance, 100 Ω (Ohm) resistance, with 2 pulses at a 2 mm (Millimeter) electrode cup, and another at 1800 V, 0.18 ms (Millisecond) pulse length, 30 s interval, with 2 pulses at a 4 mm electrode cup. The plasmids and primers for integrating the homologous templates (TransStart^®^ FastPfu Fly DNA Polymerase, TransGen) at the target sites are listed in the Supplementary Material Table S10. Genomic DNA from the parasites was extracted [37 °C for 10 min (Minutes), 56 °C for 10 min, 98 °C for 10 min] using the TransStart^®^ FastPfu Fly DNA Polymerase Extraction Kit and subsequently amplified (95 °C for 5 m; 30 cycles of 95 °C for 30 s (Second), 58 °C for 30 s, 72 °C for 1 min; 72 °C for 10 min) with a Mastercycler thermal cycler (Eppendorf SE, Hamburg, Germany).

### 4.3. Reagents and Antibodies

Rapamycin (TGI, R0097, Boston, MA, USA) was prepared as a 50 μM stock solution by dissolving it in DMSO, and a working concentration of 50 nM (1:1000 dilution) was utilized. Mycophenolic acid (Merck, M3536, Darmstadt, Germany) was mixed with anhydrous ethanol to create a 12.5 mg/mL solution, resulting in a working concentration of 25 μg/mL. Xanthine (Merck, X4002) was dissolved in 1 M NaOH to form a 25 mg/mL stock solution, with a working concentration set at 50 μg/mL. Ethamipride (Merck, 46760) was also dissolved in anhydrous ethanol to yield a 3 mM stock solution, and a working concentration of 3 μM was applied. All compounds were diluted in the culture medium to achieve the specified final concentrations for each experiment. The primary antibodies used for IFA included: anti-TY (mouse), anti-ACP (rabbit), anti-Tubulin (rabbit), and anti-IMC (rabbit) at a dilution of 1:500, sourced from Prof. Shaojun Long, along with rabbit anti-GAR12 at a dilution of 1:200, provided by Prof. Jinlei Wang from the Chinese Academy of Agricultural Sciences in Lanzhou. The secondary antibodies for IFAs comprised Alexa Fluor 488 goat anti-mouse IgG (H + L) at a dilution of 1:3000 (Invitrogen A11029, Carlsbad, CA, USA) and Alexa Fluor 568 goat anti-rabbit IgG (H + L) at a dilution of 1:3000 (Invitrogen A11036). For Western blotting, the primary antibodies included anti-TY (mouse) at a dilution of 1:10 and anti-actin (rabbit) at a dilution of 1:200. The secondary antibodies for WB were goat anti-mouse IgG (H + L) HRP conjugate at a dilution of 1:3000 (TransGen, HS201-01) and goat anti-rabbit IgG (H + L) HRP conjugate at a dilution of 1:3000 (TransGen, HS101-01), obtained from ThermoFisher Scientific (Waltham, MA, USA) and Sigma (St. Louis, MO, USA) [15].

### 4.4. Western Blots

Parasites were gathered and re-dispersed in an SDS-PAGE loading buffer (TransGen, DL101), then heated for approximately 10 min. They were separated using SDS-PAGE and subsequently transferred to a PVDF membrane via a wet transfer method (Servicebio, Wuhan, China). The membrane was then treated with 5% skim milk in TBS (Solarbio, T1082) and left to incubate overnight at 4 °C with the primary antibody. Following this, it was exposed to the secondary antibody after washing [56].

### 4.5. Indirect Immunofluorescence Assays

In this research, *Toxoplasma gondii* parasites grown on coverslips within HFF cells were treated with 4% paraformaldehyde for a duration of 15 min. They were then permeabilized using a solution of 5% BSA in PBS containing 0.2% Triton X-100 for another 15 min. Following this, the samples were blocked with PBS and 5% BSA for 30 min before being incubated with the specified primary antibodies. The samples underwent five washes with a 5% BSA solution containing 0.05% Tween-20 in PBS, followed by incubation with the appropriate secondary antibody [15]. After another five washes with the same BSA solution, they were mounted using ProLong™ Gold antifade reagent (Invitrogen, P36930) and analyzed using a confocal microscope (LabRAM Soleil, HORIBA Scientific, Palaiseau, France) with NIS-Elements Viewer software 4.60 for image capture.

### 4.6. Plague Assays

Approximately 200 tachyzoites of *Toxoplasma gondii* were counted and introduced into HFFs using freshly prepared 6-well plates. Parasites were added and removed over a 12 h period, followed by the addition and removal of rapamycin after 18 h. The cells were then cultured in DMEM supplemented with 5% FBS at 37 °C for a week. After fixation with 70% ethanol for 15 min, the samples were stained with 0.5% crystal violet for one hour, rinsed with dH_2_O, and allowed to air dry at room temperature [15]. .

### 4.7. Mice Experiment

An analysis was conducted using GraphPad Prism version 10 to assess the mortality rates of 6-week-old female BALB/C mice (18–22 g) sourced from Xinjiang Medical University. After one week of acclimatization, mice were randomly assigned to eight experimental groups (*n* = 5 per group): (1) DiCre (−Rapa); (2) DiCre (+Rapa); (3) iKD-PBGD (−Rapa); (4) iKD-PBGD (+Rapa); (5) iKD-UROS (−Rapa); (6) iKD-UROS (+Rapa); (7) iKD-UROD (−Rapa); and (8) iKD-UROD (+Rapa). Under specific pathogen-free conditions, the mice were divided into groups of five, with each group receiving an intraperitoneal injection of 100 *Toxoplasma gondii* tachyzoites, following the methodology outlined by [36]. The mice were provided with regular water until their death, while being monitored after the injection of the *Toxoplasma gondii* tachyzoites (including strains DiCre, iKD-PBGD, iKD-UROS, and iKD-UROD, each containing approximately 100 tachyzoites).

### 4.8. Replication Assay

The iKD and DiCre strains were introduced into 24-well plates that had been pre-cultured with HFF cells and allowed to grow for 12 h. Following this, rapamycin was either included or excluded from the culture. After an additional 6 h, the rapamycin was removed, and the cells were fixed using 4% paraformaldehyde for 15 min at either 48 h or 60 h after the rapamycin treatment [36]. The strains were then permeabilized with 5% BSA in PBS containing 0.2% Triton X-100, blocked with PBS and 5% BSA for 30 min, and subsequently incubated with mouse anti-TY and rabbit anti-Tubulin antibodies. This was followed by treatment with the appropriate Alexa Fluor 488 goat anti-mouse IgG (H + L) and Alexa Fluor 568 goat anti-rabbit IgG (H + L), with analysis conducted via confocal microscopy. The count of parasitophorous vacuoles (200) was recorded, noting that each could contain between 1 and 16 tachyzoites or 32 to 256 tachyzoites, depending on the phase of the parasites.

### 4.9. Statistical Analysis

Statistical analyses were performed using GraphPad Prism version 10.1.2 (GraphPad Software, San Diego, CA, USA). Data are presented as mean ± standard deviation (SD) from at least three independent experiments. Comparisons between two groups were analyzed using unpaired, two-tailed Students *t*-test. Comparisons among multiple groups were performed using one-way analysis of variance (ANOVA) followed by Tukey’s post hoc test for multiple comparisons. For two-factor comparisons involving multiple groups and time points, two-way ANOVA was applied. For survival assays, Kaplan–Meier curves were generated and compared using the log-rank (Mantel–Cox) test. A *p*-value < 0.05 was considered statistically significant (*p* < 0.05, *p* < 0.01, ** *p* < 0.001, ** *p* < 0.0001). All statistical tests and sample sizes (*n*) are indicated in the corresponding figure legends [56].

## 5. Conclusions

Research involving replication experiments has shown that three enzymes associated with the conserved heme pathway in *Sarcocystide* are crucial for the survival of the parasite during its lytic cycle. The fluorescence signals of TgGRA12 do not exhibit any statistical variation, as initially investigated regarding heme nutrition or substrate transport mechanisms. This is particularly evident in the gradual formation of large cyst-like PV, which primarily occurs around 48 h into the lytic cycle. This observation is linked to the knockdown of TgPBGD, TgUROS, and TgUROD, which connects to a cross-referenced analysis of the putative proteomes of *Toxoplasma gondii* stroma apicoplast proteins.

## Data Availability

The proteomic datasets analyzed in this study were obtained from previously published studies and are cited in the references [7,8,9]. Specifically, the proteomic data from apicoplast-less (renamed for analysis in this study: *T. gondii*^Apico^) parasites [7] are available from the Yareta (Geneva) repository under the identifier kc446nwxfvbvt5z7l2f6tayga (https://doi.org/10.26037/yareta:kc446nwxfvbvt5z7l2f6tayga); the hyperLOPIT spatial proteomics data [8] are accessible via the ProteomeXchange Consortium through the PRIDE repository under accession number PXD015269; and the APT1-TurboID proteomics data [9] are deposited in the OMIX database (China National Center for Bioinformation) with accession number OMIX002059.The newly generated data from the wet-lab experiments performed in this study are presented within the article and its Supplementary Material. The raw data are not publicly available due to [reason, e.g., institutional policy/ongoing study/privacy restrictions], but are available from the corresponding author on reasonable request.

## Supplementary Materials

The following supporting information can be downloaded at: https://www.mdpi.com/article/10.3390/ijms27188154/s1.
