# Supplementary material for: Integrated Proteomic Screening Reveals Heme Enzyme Depletion Induces Cyst-like Vacuole Formation in Toxoplasma gondii
Source: Int J Mol Sci. 2026 Sep 13;27(18):8154. doi: 10.3390/ijms27188154 (PMC13607938; doi:10.3390/ijms27188154)
Supplement: Supplementary file 1 [file ijms-27-08154-s001.zip › Supplement figure legend.pdf]

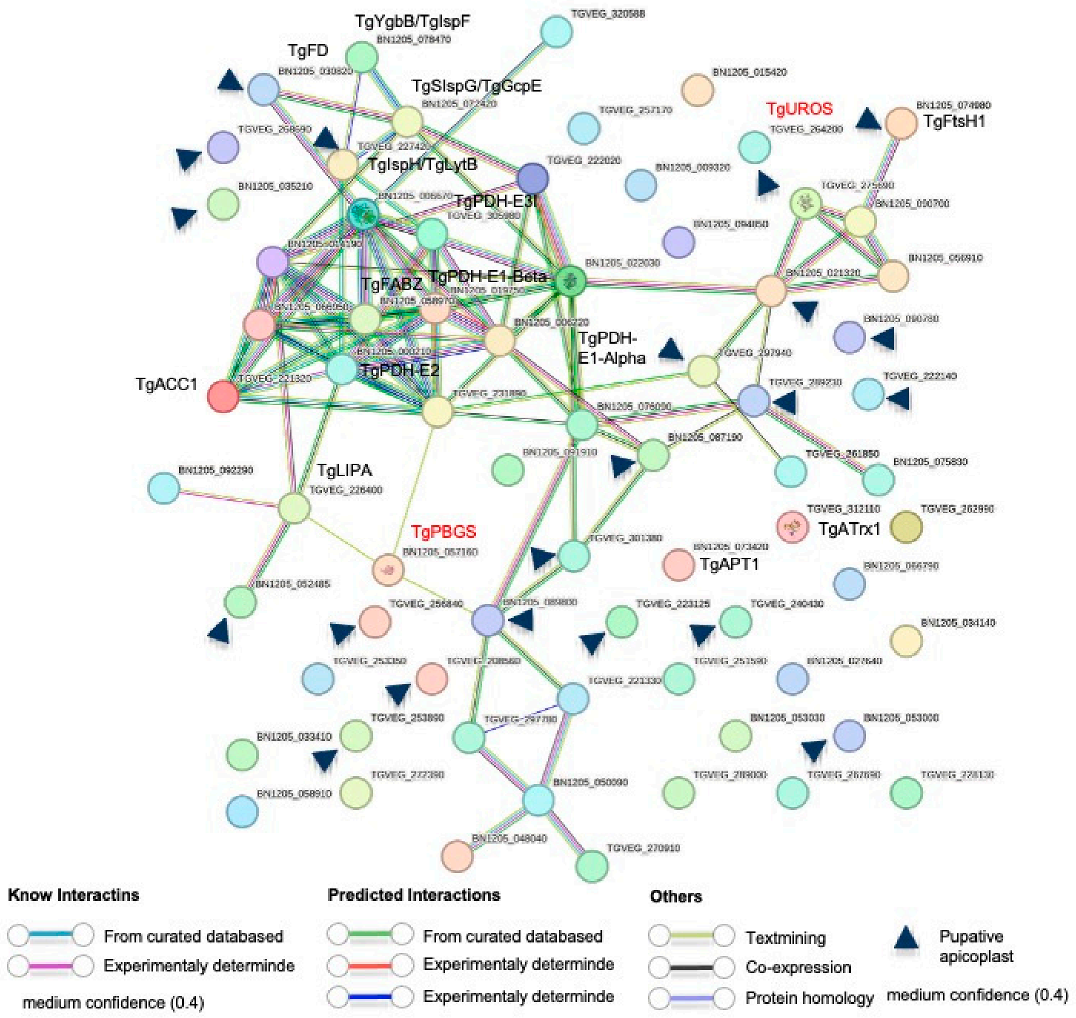

Figure S1. All 70 proteins screen up from three protein databases, and network nodes, associations and interactions through the STRING web. Which **attachment corresponds** to The **Supplement Figure protein list** and based on **Table S4**.

Protein-protein interaction network of screen apicoplast proteins (in Toxoplasma gondii or relevant organism). Network was constructed by STRING database with a medium confidence score cutoff of 0.4. Reported apicoplast proteins at centrally network have connection with each other apicoplast proteins like TgACC1 (TGVEG_221320), TgFABZ (BN1205_058970), TgIspH/TgLytB (TGVEG_227420), TgFD (BN1205_030820), and heme pathway enzymes TgPBGS (BN1205_057160), TgUROS (TGVEG_2264200). Node colors represent distinct protein clusters or functional associations. Edge colors denote interaction evidence types: Known interactions include curated database entries (cyan) and experimentally validated interactions (magenta); Predicted interactions include database predictions (green), experimental predictions (red), and other experimental evidence (blue); Other associations include text mining (yellow-green), co-expression patterns (black), and

protein homology (light purple). Triangular markers indicate subcellular localization: dark blue triangles indicate putative apicoplast proteins analysis in this study. Protein identifiers follow the TGVEG (ToxoDB) and BN1205 nomenclature systems.

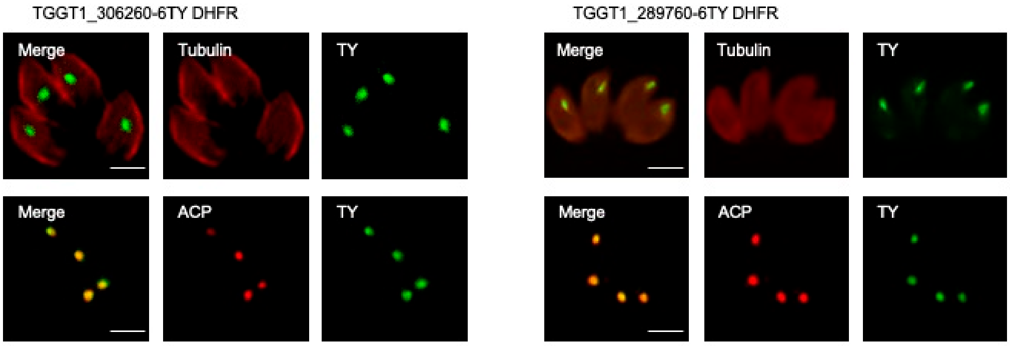

Figure S2. Indirect immunofluorescence of TGGT1_306260 and TGGT1_289760 endogenous labeled strain. Detected by anti-TY antibodies and anti-Tubulin antibodies, Scale bars = 10 μm.

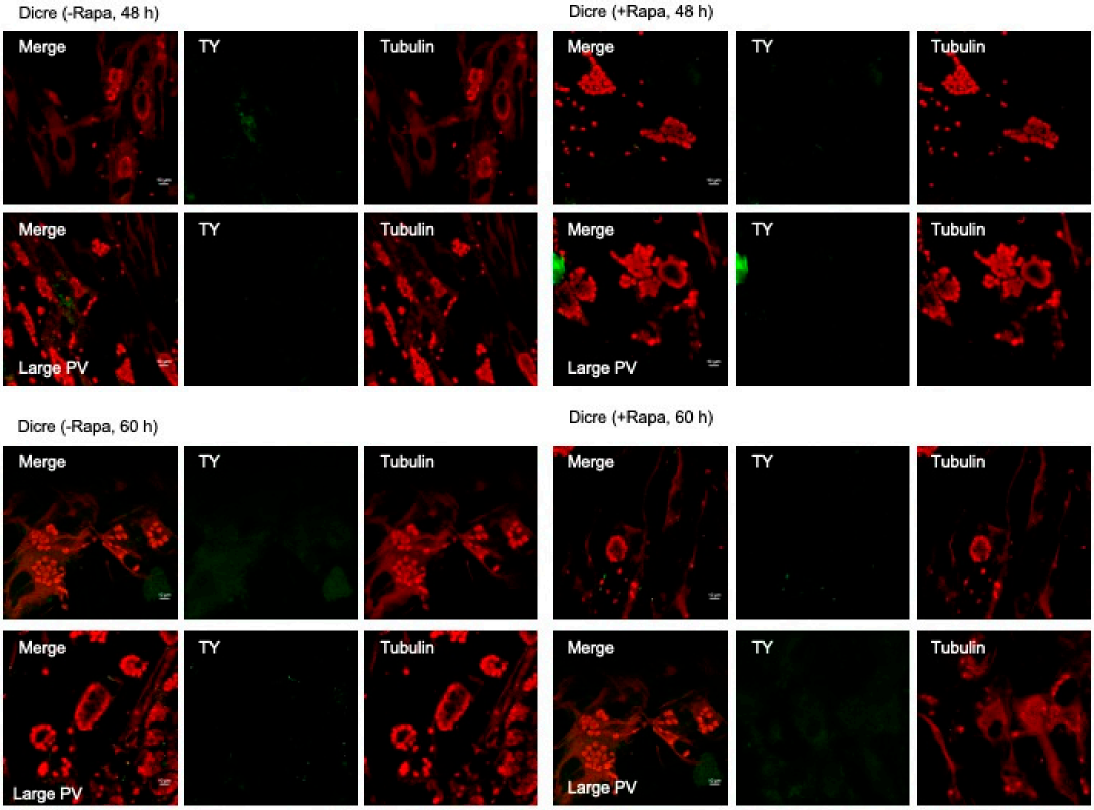

Figure S3. Indirect immunofluorescence of Dicre strain treated with rapamycin for 48 h, anti-Tubulin as parasite cytoskeleton marker.

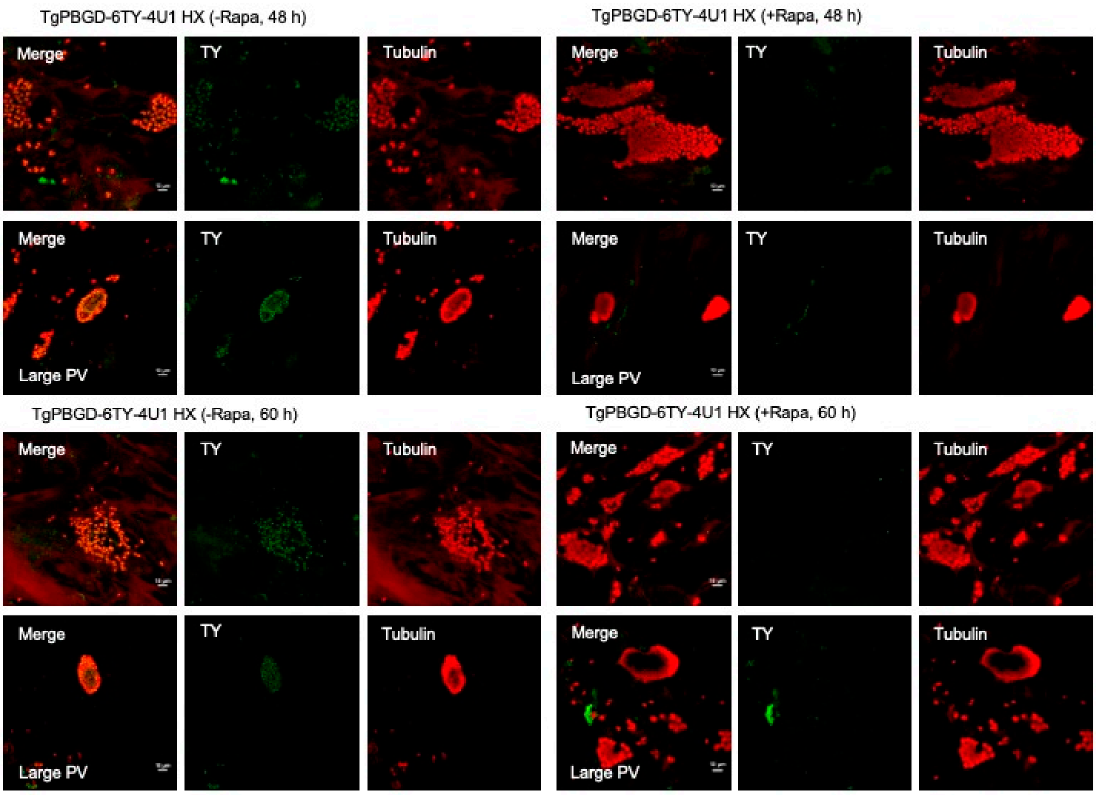

Figure S4. Indirect immunofluorescence of iKD-PBGD strain treated with rapamycin for 48 h, anti-Tubulin as parasite cytoskeleton marker.

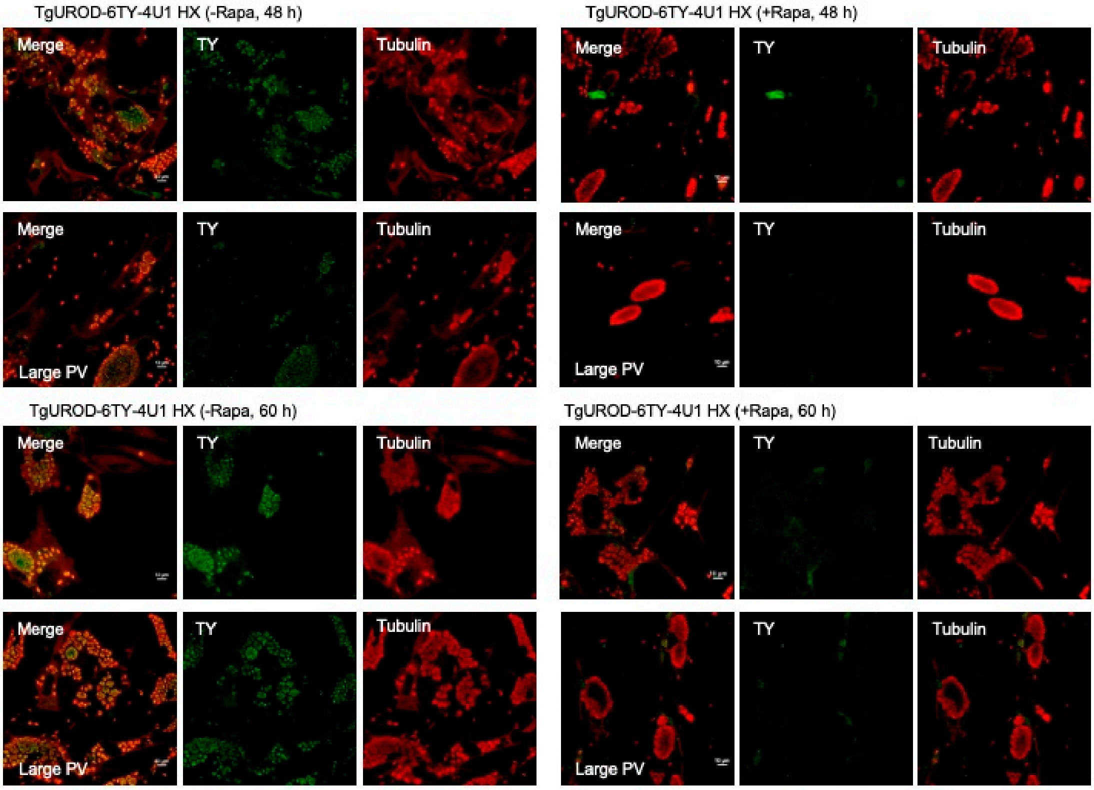

Figure S5. Indirect immunofluorescence of iKD-UROD strain treated with rapamycin for 48 h, anti-Tubulin as parasite cytoskeleton marker.
