# Supplementary material for: Integrated Proteomic Screening Reveals Heme Enzyme Depletion Induces Cyst-like Vacuole Formation in Toxoplasma gondii
Source: Int J Mol Sci. 2026 Sep 13;27(18):8154. doi: 10.3390/ijms27188154 (PMC13607938; doi:10.3390/ijms27188154)
Supplement: Supplementary file 1 [file ijms-27-08154-s001.zip › Supplement Tables S1-S9 legend.pdf]

Table S1. Detailed data organization of APT1-TurboID and HyperLOPIY-Apicoplast cross with T. gondii^{-Apico} database (putative apicoplast proteins of unchanged, increased or decreased). Which in the **attachment corresponds** to main **Figure 1 raw datas,** and had described in **Data Availability Statement** and **References**.

Table S2. Detailed data organization of T. gondii^{-Apico} database (putative apicoplast proteins of **unchanged**). Which cross with APT1-TurboID and HyperLOPIY-Apicoplast datas and shown as **Venn diagram**, in the **attachment corresponds** to main **Figure 1A-first part,** and had described in **Data Availability Statement** and **References**.

Table S3. Detailed data organization of T. gondii^{-Apico} database (putative apicoplast proteins of **increased**). Which cross with APT1-TurboID and HyperLOPIY-Apicoplast datas and shown as **Venn diagram**, in the **attachment corresponds** to main **Figure 1-the second part**, and had described in **Data Availability Statement** and **References**.

Table S4. Detailed data organization of T. gondii^{-Apico} database (putative apicoplast proteins of **decreased**). Which cross with APT1-TurboID and HyperLOPIY-Apicoplast datas and shown as **Venn diagram**, in the **attachment corresponds** to main **Figure 1A- the third part** and **Evenn network Figure 1B**, **or** in the **attachment corresponds to Supplement Figure protein list** to shown in **Protein-protein interaction network- figure S1,** and had described in **Data Availability Statement** and **References**.

Table S5. Screened the detailed data organization of T. gondii^{-Apico} database (putative apicoplast proteins of **decreased**). Which cross with APT1-TurboID and HyperLOPIY-Apicoplast datas and shown as **screened diagram**, in the **attachment corresponds** to main **Figure 1B- the second part Evenn network**, and had described in **Data Availability Statement** and **References**.

Table S6. 23 the putative apicoplast that form of a presentation as **Protein-protein interaction network** in the **attachment corresponds to main Figure 2,** and had described in **Data Availability Statement** and **References**.

Table S7. The data of homology analysis of heme biosynthesis pathway enzymes in apicomplexan, in the **attachment corresponds** to main **Figure 3.**

Table S8. The data of replication of heme biosynthesis pathway enzymes that formation of parasitophorous vacuoles at different stage, in the **attachment corresponds** to main **Figure 4E and Figure 5B.**

Table S9. The data of probability of death to the viability of parasite, in the **attachment corresponds** to main **Figure 4D.**

Supplement Figure protein list

All 70 proteins screen up from three protein databases (**attachment corresponds to Figure S1** and based on **Table S4**), and network nodes, associations and interactions through the **STRING** web. Could be screen 23 the putative apicoplast (**attachment corresponds to Table S6**) that form of a presentation as **Protein-protein interaction network** in the **attachment corresponds to main Figure 2,** and had described in **Data Availability Statement** and **References**.
