# Supplementary material for: Integrated Proteomic Screening Reveals Heme Enzyme Depletion Induces Cyst-like Vacuole Formation in Toxoplasma gondii
Source: Int J Mol Sci. 2026 Sep 13;27(18):8154. doi: 10.3390/ijms27188154 (PMC13607938; doi:10.3390/ijms27188154)
Supplement: Supplementary file 1 [file ijms-27-08154-s001.zip › Supplementary Material Table S10.pdf]

Supplementary Material Table S10: Primer, Plasmid, Antibody and Strains.

Primer	Sequences	Source
UROS-sgRNA	TAAATGGGGATGTCAAGTTACAACGTGTTCGCTCCTC GTGTTTTAGAGCTAGAAATAGC	-
UROS-L	ACACGCGATAGGCTCGAGCCCTAGAGAGACGCCGAA GAAGgctagcaagggtcgg	-
UROS-T	AAAGAAATCTGCTACCTAAAGAGTCGTTCTGGAACCC ACGtcccagtcacgacgttg	-
UROD-sgRNA	TAAATGGGGATGTCAAGTTAGGAACGAGTGACTTACT GGGTTTTAGAGCTAGAAATAGC	-
UROD- L	TCGAGAACTCCGCGGGGAGGGCCAAGATACCTCAAC AGTTgctagcaagggtcgg	-
UROD- T	TGTCTTCACAGAGCGTCTTGTGTAAACGCGTGCTCCC CCAtcccagtcacgacgttg	-
PBGD-sgRNA	TAAATGGGGATGTCAAGTTAAACGACGTTGCTCACCT GGGTTTTAGAGCTAGAAATAGC	-
PBGD-L	AAAGCATGTCACTGAAGGTTGGAAGAATTTGAAAAC GTTGgctagcaagggtcgg	-
PBGD-T	GCGACGTAGTTGACGATCGGTCGTGTCTGTACACCCG CCAtcccagtcacgacgttg	-
TGGT1_306 260-sgRNA	TAAATGGGGATGTCAAGTTAGTCTTGCACTCTGGGTAC AGTTTTAGAGCTAGAAATAGC	-
TGGT1_306 260-L	CCTTCAAGAACAAAACCGGGAAAGGCCTGTGCAGAG CAAAgctagcaagggtcgg	-
TGGT1_306 260-T	CGACAATTCCCGACCGCAATGCAATTTTTCATAACCTT GTtcccagtcacgacgttg	-
TGGT1_289 760-sgRNA	TAAATGGGGATGTCAAGTTGCCCAGATGCTTTGACTTG GGTTTTAGAGCTAGAAATAGC	-
TGGT1_289 760-L	GAGGACGCAAAAGTTTGTCGGTGGTCATCTTGTGAGG CCCgctagcaagggtcgg	-
TGGT1_289 760-T	TCTAGTCATCACTCTTTTCGCAGACCCAAGAAAACCG CCAtcccagtcacgacgttg	-
	Cell strain	
	HFF-1 line,	ATCC SCRC-1041
Plasmid	Plasmid	
	P-SAG1::Cas9-U6:sgRNA	Prof. Shaojun Long
	P-6Ty-DHFR	Prof. Shaojun Long
	P-linker-6ty-loxp-3UTR-loxp-4U1-HXGPRT	Prof. Shaojun Long

Antibody	Antibody	
	Anti-TY (mouse)	Prof. Shaojun Long
	Anti-Tubulin (rabbit)	Prof. Shaojun Long
	Anti-GAR12 (rabbit)	Prof. Jinlei Wang
	Anti-actin (mouse)	Prof. Shaojun Long
	Anti-IMC (rabbit)	Prof. Shaojun Long
	Goat anti-mouse IgG (H+L) HRP conjugate	TransGen, HS201-01
	Goat anti-rabbit IgG (H+L) HRP conjugate	TransGen, HS101-01
	Alexa Fluor 488 goat anti-mouse IgG (H+L)	Invitrogen A11029
	Alexa Fluor 568 goat anti-rabbit IgG (H+L)	Invitrogen A11036
Strains	Strains	
	RHΔ*hxgprt*Δ*ku80*	Prof. Shaojun Long
	RHΔ*hxgprt*Δ*ku80*-T2A-DiCre	Prof. Shaojun Long
	RHΔhxgprtΔku80-T2A-DiCre/PBGD-6TY-4U1 HXGPRT	This study
	RHΔhxgprtΔku80-T2A-DiCre/UROS-6TY-4U1 HXGPRT	This study
	RHΔhxgprtΔku80-T2A-DiCre/UROD-6TY-4U1 HXGPRT	This study
	RHΔ*hxgprt*Δ*ku80*/TGGT1_306260-6TY DHFR	This study
	RHΔ*hxgprt*Δ*ku80*/TGGT1_289760-6TY DHFR	This study
